# Challenges and Recommendations to Recruiting Women Who Do Not Adhere to Follow-Up Gynecological Care

**DOI:** 10.4236/ojpm.2014.43017

**Published:** 2014-03-20

**Authors:** LaShawn Wordlaw-Stinson, Sierra Jones, Shaneese Little, Laura Fish, Adriana Vidal, Jennifer S. Smith, Cathrine Hoyo, Patricia G. Moorman

**Affiliations:** 1 Department of Public Health Education, North Carolina Central University, Durham, USA; 2 Duke University Medical Center, Durham, USA; 3 University of North Carolina-Chapel Hill, Durham, USA

**Keywords:** Non-Adherence, Patient Compliance, Cervical Cancer, Cervical Intraepithelial Neoplasia, Cancer Screening, Abnormal Pap Smear, Abnormal Pap Test, HPV, Patient Navigation

## Abstract

**Purpose:**

Non-adherence to recommended follow-up visits after an abnormal cytological finding is associated with poorer outcomes and higher health care costs. The purpose of this paper is to describe the challenges when examining reasons for non-adherence to cervical cancer screening follow-up and to discuss the recommendations to overcome those challenges.

**Methods:**

We conducted a telephone survey with two subgroups of women: 1) those which adhered to recommended follow-up care after an abnormal Pap test, and 2) those which did not adhere.

**Results:**

The follow-up accrual among non-adherent women lagged behind that of adherers. We were able to contact and conduct a survey with 51% of the adherers and 26% of the non-adherers. The challenges in studying non-adherent women were related to several distinct factors: 1) the definition of non-adherence, 2) the availability of alternate contact information, 3) the amount and type of financial incentives, and 4) the availability of staffing. We describe strategies employed to increase the accrual of non-adherent women.

**Discussion:**

This paper describes four recommendations that may play a role in understanding and reducing non-adherence to follow-up gynecological care.

## 1. Introduction

If cervical cancer is nearly 100% preventable, why does the American Cancer Society estimate that 12,340 women will be newly diagnosed and 4,030 will die this year alone in the United States? [1]. In addition, it is well-established that minority women suffer disproportionately from cervical cancer with higher incidence and mortality rates among Hispanic and Non-Hispanic Black women [2]. Although cervical cancer mortality is a complex problem, three factors that influence it are: 1) regular utilization of the Pap test, 2) accuracy of the Pap test results, and 3) timely follow-up for abnormal Pap test results.

The Pap test is well-integrated into medical practice and screening coverage rates are estimated to be greater than 80% in the United States [3]. Although screening maintenance remains as an important factor for some women, the lack of timely follow-up of abnormalities is a significant problem as it is related to late-stage diagnosis and low survival [4]–[7].

Evidence from a recent systematic review indicates that approximately 15% – 18% of women who undergo Pap testing each year require additional follow-up [8]–[11].

As a part of a larger study investigating genetic predictors of progression of cervical lesions (Cervical Intraepithelial Neoplasia Cohort Study (CINCS)), we explored reasons associated with non-adherence to recommended follow-up evaluation after an abnormal Pap test result. We recognized that it is very challenging to address this problem because the same characteristics and behaviors that are associated with non-adherence may also make these women less likely to agree to be a part of a research study. However, this research has implications for designing intervention studies to improve adherence to follow-up care, and for implementing systems-level procedures for health care facilities/clinical settings that promote adherence. The purpose of this paper is to describe the challenges that we encountered while exploring reasons for non-adherence and some recommendations which we employed for overcoming those challenges.

## 2. Methods

### 2.1. Participants

To be eligible for the CINCS study, women had to: 1) have a recent abnormal Pap test, 2) have a uterus (no hysterectomy), and 3) be between the ages of 21 and 79 years of age. As an adjunct to the CINCS study, we conducted a follow-up telephone survey with a subset of adherers and non-adherers. Adherers are defined as women who showed up for their scheduled follow-up appointment and non-adherers are women who did not show up.

### 2.2. Instrumentation

The participants were asked a brief set of questions consisting of demographic characteristics, knowledge and attitudes about cervical cancer and the human papilloma virus (HPV) infection. These data were analyzed in SAS as previous described. The Institutional Review Boards at North Carolina Central University and Duke University approved this study.

## 3. Results

In October 2011 we had completed telephone interviews with 53 adherers and 34 non-adherers. By December 2011, we had identified challenges and implemented solutions to increase the enrollment of the non-adherers. When the study concluded in May of 2012 a total of 184 women consented to participate in the telephone interview; 92 adherers and 92 non-adherers. The next section outlines the challenges and recommendations based on our efforts to obtain comparable enrollment.

### 3.1. Challenge & Recommendation 1: Definition of Non-Adherence

The first challenge was the definition of non-adherence. Health professionals agree that non-adherence is a complicated patient behavior with definitions that vary across studies and disciplines [12]. The most common definition is from Haynes *et al*. which simply describes adherence as whether patients follow the instructions of health care providers [13]. Specifically, for examining barriers to follow-up for abnormal Pap tests, non-adherence occurs within a time period determined by the health care facility considering the guidelines [14]. Our study initially defined non-adherence as missing the first scheduled follow-up appointment. However, two scenarios altered this definition. Scenario 1: Women who did not show up for their first scheduled appointment were appropriately displayed on the listing of non-adherent women. However if they re-scheduled and adhered to a subsequent appointment, they were not truly non-adherent. Thus non-adherence was a time-dependent construct. Scenario 2: During the telephone interviews, a few women stated that they *had* received follow-up care at another healthcare facility because they relocated. This indicates that some women initially thought to be “non-adherent” *had* received care, but we were unable to verify this information without access to medical record data. In future observational and intervention studies, we recommend defining non-adherence as a time related variable. For example, women who did not return for a follow up appointment in *x* number of months rather than as missing one (the first) scheduled appointment. We also recommend verifying self-report information with another source when possible. For researchers who design intervention studies, considering how non-adherence is defined in similar settings is beneficial for comparing results across studies. For programs the definition of non-adherence is tied to clinical recommendations, therefore it is important to have confirmation that the patient has been notified of the abnormal result and to ensure that they understand and are able to take necessary next steps toward follow-up care in a timely manner.

### 3.2. Challenge & Recommendation 2: Alternate Contact Information

The primary method of contact was through the telephone number listed in the health system scheduling appointment log. A challenge we encountered was disconnected telephone numbers among the non-adherers. Approximately 25% – 30% of the call attempts resulted in disconnected telephone numbers for non-adherers, whereas very few disconnected telephones were encountered for adherers. A reason for this difference is that the adherers who were also enrolled in the larger CINCS study had their contact information updated. Anecdotal accounts suggest that some women of lower socioeconomic status use pre-paid cell phones, resulting in more disconnected or changed phone numbers. We recommend that in addition to updating patient contact information at every medical care encounter clinic staff ask for alternate contacts such as relatives and trusted friends who can serve as another notification cue. In the absence of a computerized tracking database specifically designed to monitor and follow-up, in our experience, women who did not provide alternate contact information represented a loss to the study. Researchers and program administrators should plan ahead for the possibility that follow-up care is needed. Informing patients about the benefits of providing alternate contact information supports timely notification which may be positively associated with timely care.

### 3.3. Challenge & Recommendation 3: Amount and Type of Financial Incentives

As in many studies, incentives to cover participant time and effort may positively influence recruitment and retention. Financial incentives may be particularly important in hard-to-reach populations. Previous research indicates that economic vouchers and other financial incentives improve follow-up rates for abnormal Pap test results [15]. For this study, the effort to get a woman on the phone was an obstacle with the high number of disconnected phone numbers, therefore to increase the odds of her participation the amount and type of incentive was re-considered. Prior to December 2011, the incentive was the same for adherers and non-adherers to complete the telephone survey. The participants could choose either a $10 retail gift card or a $10 check. It is worthwhile to note that the $10 check option was not as desirable as the gift card. For women of lower socioeconomic status without checking accounts, incentives in the form of a gift card seem to be acceptable. Two changes were employed regarding incentives. The first change is that the incentive for non-adherers was increased to $25. The second change is that retail gift card was changed to a VISA gift card allowing participants the flexibility to spend it wherever they wanted. A general gift card rather than one for a specific merchant seemed to be a better option. For non-adherent women who may not have been highly motivated to complete the telephone survey, we believe that changing the amount and type of the financial incentive played a role in the rise in accrual rates. The challenge of determining the appropriateness of the financial incentive may be a signal to researchers to consider the ongoing relationship between economics and compliance.

### 3.4. Challenge & Recommendation 4: Staffing

The last challenge is related to staffing, which we realize may be limited by a program’s budget. Still there are considerations that could make a difference. In our case, recruiting women who do not adhere to recommended follow-up care required more effort. For example, the challenge of the disconnected phone numbers resulted in approximately 350 call attempts to non-adherers to obtain a subsample of 92, whereas for adherers less than 150 were called for subsample of 92. The research study employed up to 4 research assistants but the key was calling at different hours of both weekday and weekends to recruit the sample of non-adherent women. A primary advantage to having more than one person make calls to this hard-to-reach population is that more days and hours in a day can be covered, which may be especially important for women who are not available by phone during standard business hours. Having multiple staff make calls also facilitated making calls soon after the missed follow-up appointment theoretically increasing the probability of a functional telephone number by capturing the window of opportunity before it may become disconnected. For researchers when there is an opportunity to re-allocate budgetary resources prioritizing staffing is essential for any project particularly for one that faces challenging accrual. Trained entry-level health educators rather than nurses are ideal to track and monitor follow-up.

## 4. Discussion

We share our lessons and provide recommendations based on our experience with recruiting women who do not return for follow-up care after being notified of an abnormal Pap test result. Even with evolving recommendations such as those indicated in 2012 from the American Society for Colposcopy and Cervical Pathology (ASCCP), to use HPV/cytology co-testing every five years for women age 30 and older, the timely return for the evaluation of abnormal outcomes is crucial. HPV/cytology co-testing increases accuracy to improve the early detection of cervical cancers [16]. Still, women must return for further evaluation and if necessary, treatment. On the other hand, it is also important for clinicians, program administrators, and researchers to avoid putting all non-adherers into one category. Khanna & Phillips (2001: 124) identified three subgroups of non-adherers: 1) patients who were aware of their Pap test results and the prescribed care plan, but chose not to adhere, 2) patients who were interested in adhering to the care plan, but were unable, and 3) patients who followed their provider’s recommendation, but these recommendations deviated from the standard follow-up guidelines [17]. Theoretically each subgroup of non-adherers will likely require different intervention strategies. More research is needed in this area.

The issues that we uncovered when trying to understand reasons for non-adherence similarly apply to those faced by health care providers as they interact with women undergoing cervical cancer screening. For example, the challenges that we describe are likely those that health care providers encounter when trying to insure that their patients receive timely follow-up for abnormal Pap test results. The recognition that there are at least three sub-groups of non-adherers suggests that health care providers must work to optimize communication with their patients to gain a clear understanding of concerns by identifying those that may affect their willingness to adhere to recommended care. Although we know that health care providers do encourage patients to return for follow- up care, the challenges that we faced in this study offer opportunities to re-think how to encourage women to return for follow-up care whether they “chose” not to or are “unable” to adhere. The heart of these recommendations is the need for improved communication between health care providers and to empower patients to make informed decisions about their care with adequate support and resources to alleviate concerns that may otherwise delay necessary treatment for abnormal Pap test results.

The challenge of incorrect or disconnected telephone numbers suggests that health care providers must understand that some patients change phone numbers and addresses frequently. To insure that there is reliable contact information, we recommend that there are multiple ways to contact the patient to report results, discuss results, respond to concerns, and facilitate timely follow-up care. This may include alternate telephone numbers, e-mail addresses, or even the telephone number of a trusted relative or friend. We acknowledge confidentiality concerns and caution that the content of these messages must comply with HIPAA regulations. Previous research has found that when there is a functional number, then using the telephone protocol that includes four call attempts is beneficial for study enrollment [18].

The challenge of determining the incentive suggests that health care providers should consider financial barriers that may influence follow-up for both insured and uninsured patients. Oftentimes it is thought that only patients who do not have health insurance coverage are non-adherent, however, although they may represent the majority of cases, it is also true that those who have health insurance also engage in the lack of follow-up behavior as additional services may not be fully covered and the estimate of the out-of-pocket cost for the follow-up care is not known until afterwards. Therefore, we recommend that health care providers consider including case management services related to financial costs to clarify and respond to patients’ stated or unstated concerns that may impede their ability to keep their scheduled appointments. Previous research suggests that vouchers, which pay a portion of the cost of care, may have a moderate impact on loss to follow-up [19]. In addition, if financial concerns prevent patients from obtaining crucial follow-up care, the provider may be able to direct patients to institutional or public health resources to assist them with paying for care. We believe strongly that a letter communicating the need to schedule a follow-up appointment without any mention of financial resources and/or assistance does not support adherence to care for those having concerns about cost.

The challenge of utilizing staff who work beyond standard daytime working hours, suggests that clarifying the importance of the follow-up coupled with providing convenient clinic hours may increase the likelihood of patients returning for follow-up care. The need for extended clinic hours is likely related to socioeconomic factors, in which some women working in hourly positions without paid sick time may not be able to take time off for medical appointments without losing income. However, implementing extended hours must be thoroughly evaluated by the clinical practice to determine the most appropriate solution. Previous research indicates that extended hours may refer to weekdays only, weekends only, or both. Extended hours can improve adherence if they are the hours of the greatest need, using staggered scheduling to avoid staff “burnout”, and having staff with a mix of professional skills who can respond to the scope of services [20].

Our experience in trying to study reasons for non-adherence shows that inadequate follow-up can sometimes happen when health care providers and patients do not communicate about the abnormal Pap test results in a manner that supports an ongoing patient-provider partnership. In the most extreme situation, which fortunately was an uncommon occurrence (<1%), some of the non-adherent women reported during the telephone survey that they were not aware that they had an abnormal Pap test result. For the larger group of women who were aware of the abnormal result, the persistent high rate of non-adherence to recommended follow-up testing suggests that breakdowns in communication between health care providers and patients may be frequent occurrences.

In addition to the recommendations offered here, we propose that women should be informed of possible outcomes of the Pap test (e.g., normal finding, requiring no additional follow-up until the next scheduled screening, abnormal finding that requires further evaluation, or an abnormal finding requiring treatment) at their Pap test appointment, so that they understand the possible scenarios and perhaps preview navigating the next steps. Ideally, this dialogue would establish or build rapport between the health care provider (or patient navigator or health educator) and the patient. The dialogue would focus on several key points such as the best method for the patient to receive screening test results, alternate contacts information, and access to other professionals who may be helpful such as nurses who have the medical knowledge to address concerns that arise during all phases of the Pap test experience (before the test, after the test, scheduling the next appointment according to the ASCCP guidelines or referral for further evaluation and/or treatment, if applicable). We believe that the time investment at the initial Pap test encounter will set the tone for an ongoing partnership between the health care provider and the patient that will not only promote informed decisions but also positively influence follow-up and increase timely medical care.
